# Femoral neck fracture osteosynthesis by the biplane double-supported screw fixation method (BDSF) reduces the risk of fixation failure: clinical outcomes in 207 patients

**DOI:** 10.1007/s00402-017-2689-8

**Published:** 2017-04-08

**Authors:** Orlin Filipov, Karl Stoffel, Boyko Gueorguiev, Christoph Sommer

**Affiliations:** 1 Vitosha Hospital, Simeonovsko shose str.108-B, 1700 Sofia, Bulgaria; 2 Cantonal Hospital Baselland, University Basel, Basel, Switzerland; 3 AO Research Institute Davos, Davos, Switzerland; 4 Cantonal Hospital Graubuenden, Chur, Switzerland

**Keywords:** BDSF, Biplane, Femoral neck fracture, Fixation, Osteosynthesis, Hip fractures

## Abstract

**Introduction:**

Osteosynthesis of femoral neck fractures is related up to 46% rate of complications. The novel method of biplane double-supported screw fixation (BDSF; Filipov’s method) offers better stability using three medially diverging cannulated screws with two of them buttressed on the calcar. Biomechanically, the most effective component is the distal screw placed at steeper angle and supported on a large area along the distal and posterior cortex of the femoral neck following its spiral anterior curve. Thereby, BDSF achieves the strongest possible distal-posterior cortical support for the fixation construct, which allows for immediate full weight-bearing. The aim of this study was to evaluate the outcomes from the first 5-year period of BDSF clinical application.

**Materials and methods:**

Subject of this retrospective study were 207 patients with displaced Garden III–IV femoral neck fractures treated with BDSF. Three 7.3-mm cannulated screws were laid in two medially diverging oblique planes. The distal and the middle screws were supported on the calcar. The distal screw had additional support on the posterior neck cortex.

**Results:**

The outcomes in 207 patients were analysed in 29.6 ± 16.8 months follow-up. Bone union occurred in 96.6% of the cases (males 97.6%, females 96.4%, *P* = 0.99). Rate of nonunion was 3.4%, including fixation failure (2.4%), pseudoarthrosis (0.5%) and nonunion with AVN (0.5%). Rate of AVN was 12.1% (males 4.8%, females 13.9%, *P* = 0.12). Modified Harris hip score was 86.2 ± 18.9 (range 10–100), with no significant difference between genders, *P* = 0.07. Older patients were admitted with significantly more comorbidities (*P* = 0.001), and on follow-up they were significantly less mobile (*P* = 0.005) and had significantly more difficulties to put socks and shoes on (*P* < 0.001).

**Conclusions:**

By providing additional cortical support, the novel BDSF method enhances femoral neck fracture fixation strength.

## Introduction

Osteosynthesis of femoral neck fractures is related up to 46% rate of complications [1, 2]. While the late avascular necrosis (AVN), ranging from 9 to 32%, depends on various biological and surgical factors, the other common complication—fixation failure, rating between 9 and 30%—is mainly due to insufficient fixation strength in osteoporotic bone [3–7]. The latter could be reduced by optimizing the primary stability of the internal fixation construct.

**Fig. 1:**
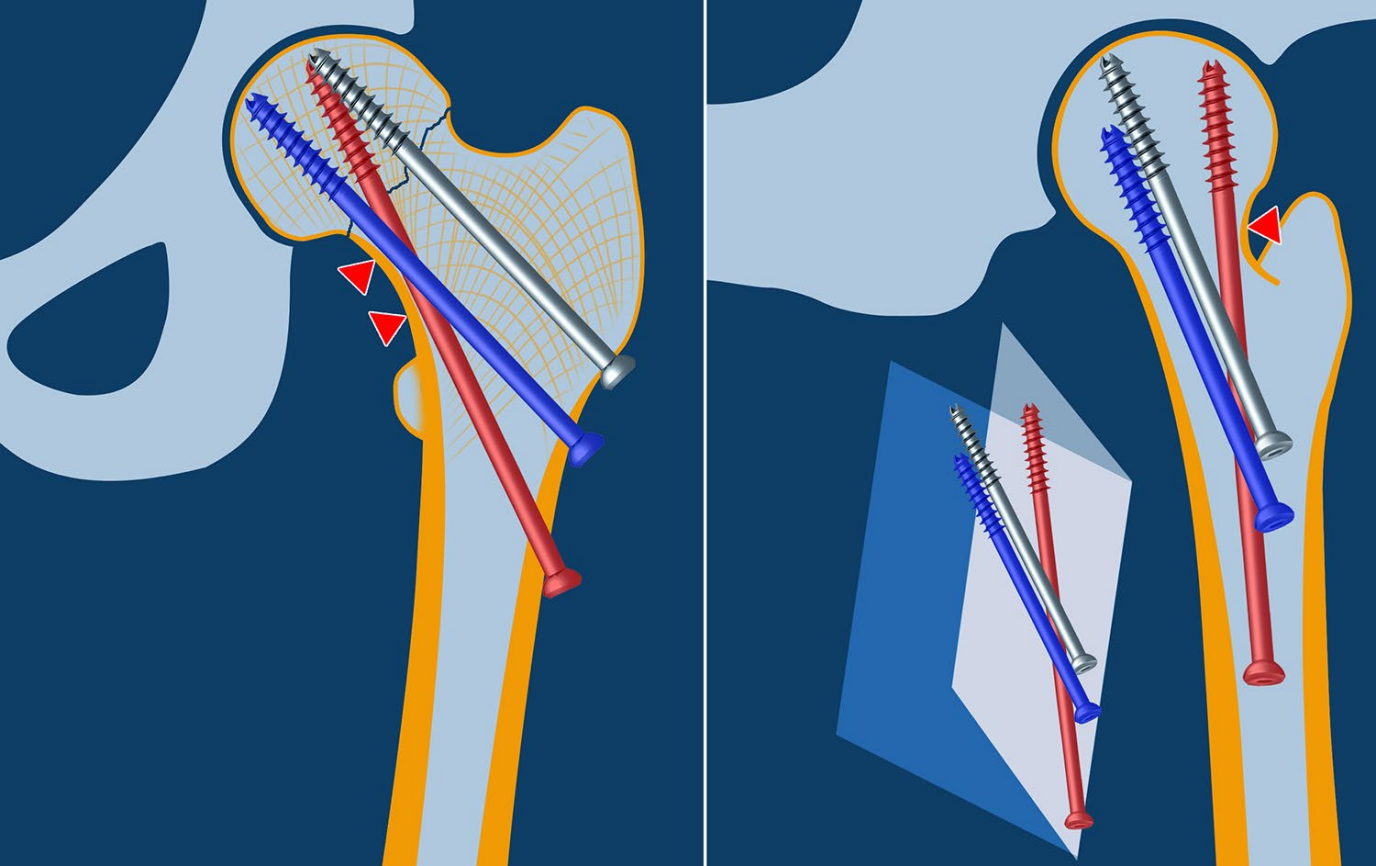
Schematic representation of the BDSF method. The distal screw (*red*) is placed in the dorsal oblique plane, whereas the middle (*blue*) and proximal screw (*grey*) are oriented in the anterior oblique plane. The distal and the middle screws are calcar-buttressed with coronal inclinations of 150°–165° and 130°–140°, respectively. Each of these screws is placed with the following two supporting points (pivots) in the distal fragment: the medial supporting point on the distal femoral neck cortex and the lateral supporting point at the screw-entry point into the lateral diaphyseal cortex. The distal screw has an additional third medial supporting point on the posterior femoral neck cortex. The three medial supporting points are indicated with* triangles*

The recently introduced novel method of biplane double-supported screw fixation (BDSF; Filipov’s method) provides improved cortical screw support and increased fixation strength [8–10]. The concept of biplane positioning makes it feasible to place three cannulated screws at steeper angles to the diaphyseal axis with entry points located much more distally within the thicker cortex of the proximal diaphysis, thus improving their beam function and cortical support. The three screws are laid in two vertical oblique planes that medially diverge toward the femoral head on lateral view (Fig. 1). BDSF implements two calcar-buttressed screws, oriented in different coronal inclinations and intended to provide sufficient stability during various physical activities. Their medial supporting points are located 10–20 mm apart, thereby distributing the axial load over a larger cortical area. Moreover, achieving posterior cortical support using an obtusely placed screw improves construct resistance to anteroposterior (AP) bending forces [11].

The aim of the present retrospective case study was to evaluate the clinical outcomes from the first 5-year period of patients’ treatment with the novel BDSF method for femoral neck fracture fixation. This study was approved by the institutional Ethical Committee.

## Materials and methods

### Patients

Subject of our retrospective study were all 207 patients with displaced fractures of the femoral neck Garden III (15 patients) and IV (192 patients) treated in our institution with BDSF in the 5-year period 2008–2012 and with a minimum follow-up period of 12 months, including all complications. The patients gave informed consent. They denied and/or were unfit for arthroplasty. Fractures Garden I and II and Pauwels type III were excluded.

### Treatment

In the BDSF procedures, we used three 7.3-mm self-tapping partially threaded (length 32 mm) steel cannulated screws (DePuy Synthes, Zuchwil, Switzerland). All patients received perioperative antibiotic prophylaxis and were given low-molecular-weight heparin until the 35th day post operation. Two surgeons familiar with the BDSF method operated the patients within 21.5 ± 19.5 h (mean ± standard deviation, SD) after admission.

Indications for application of BDSF in our practice are fractures of the femoral neck from I to IV stage by Garden, which are generally considered to meet the indications for internal fixation based on accepted clinical algorithms [12, 13]. Accordingly, BDSF can be applied for patients younger than 65 years, for high-demand patients aged more than 65 years without preexisting pathology in the hip joint, for non-ambulatory low functioning patients unfit for arthroplasty, as well as for all patients with non-displaced femoral neck fractures. Fractures of Pauwels type III are contraindicated if they pass laterally of the midcervical line. The choice of treatment approach—arthroplasty or internal fixation—is also consistent with the patients’ wish in the informed consent. All patients underwent spinal anaesthesia during operation.

### Reduction

Closed reduction by mild traction, slight abduction and internal rotation of the limb, or reduction by Leadbetter was applied, with the patient in supine position on the fracture table [14]. Aiming to achieve anatomical reduction, the following criteria for acceptable reposition were set: no varus, maximum displacement of 2 mm and valgus alignment of 0°–15° on AP view; maximum displacement of 2 mm while allowing up to 20° ventral and 10° dorsal angular displacement on lateral view [15]. Open reduction, through Watson–Jones approach, was used in two cases (0.97%).

### Surgery

The following surgical BDSF technique is well described in previous studies [8, 9].

#### Approach

A straight lateral incision is performed, starting at the level of the lower border of the greater trochanter, with a distal length of 6–10 cm. Following a direct lateral transmuscular approach, a stripping of the periosteum of the lateral diaphysis over a distance of 6–7 cm is performed.

#### Placement and positioning of guiding wires

First, we lay the guiding wire for the distal cannulated screw. Its entry point is placed 5–7 cm distally from the lower border of the greater trochanter, in the lateral surface of the stripped-off diaphysis. The wire is inclined at an angle of 150°–165° towards the diaphyseal axis and directed posterior–proximal, so that after it touches onto the curve of the distal femoral neck cortex (the “calcar”) tangentially on AP view, the wire goes into the dorsal third of femoral head and gets in contact with the posterior neck cortex (on lateral view).

The middle guiding wire is placed second. The entry point, depending on the caput-collum-diaphyseal (CCD) angle, is at 2–4 cm proximally from the distal wire. This wire is inclined at an angle of 130°–140° towards the diaphyseal axis and is directed anterior–proximal, so that after it touches onto the calcar tangentially, the wire goes into the frontal one-third of the femoral head (on lateral view) and into the distal one-third of the femoral head (on AP view).

Then, we place the proximal guiding wire, with its entry point at 1.5–2.0 cm proximally from the middle wire and parallel to it. The latter wire goes into the front one-third of the femoral head (on lateral view) and into the proximal one-third of the femoral head (on AP view).

#### Insertion of screws

Measurement of the screw lengths and drilling with a 5-mm cannulated reamer follow. The middle and proximal screws are placed first because they are perpendicular to the fracture surface. Before placing the middle and distal screws, we overdrill their holes in the lateral cortex using a 7-mm cannulated reamer, where a bone tap is difficult to be used. Next, we release the foot traction, and by gentle hammering on a plastic impactor on the diaphyseal cortex, impaction of the fracture with an additional tightening up of the screws follows. Finally, the distal screw is placed. Although the sequence of insertion of the three screws is very important, the sequence of insertion of their guiding wires is not of such importance. The guiding wire easily changes its initial direction when passing through the thick diaphyseal cortex, and therefore, its tip is guided into the desired direction by hand with the help of a cannulated instrument.

All three screws are inserted less than 5 mm subchondrally, and no screw is placed in the central zone of the femoral neck on lateral view.

No capsulotomy was performed in all cases (except in two cases requiring open reduction). Patients’ Röntgen radiation (X-ray) time during operation was 0.25 ± 0.05 min. No complications occurred during the surgery.

### After-treatment

The patients were mobilized immediately after surgery and encouraged to full weight-bearing, without limitations in the range of motion. However, younger patients, aged 55 years or less, were advised for only partial weight-bearing (30 kg) during the first 8 weeks post operation because of their dense bone not allowing increase of the frictional stability at the fracture site by intraoperative impaction.

### Data acquisition

All patients were appointed to postoperative examinations after 2, 3, 6, 12, 24 months and later. During the follow-up examinations, the healing process was documented by conventional radiographs and recording of the clinical results using the modified Harris hip score (Harris HS) questionnaire [16]. Gender, age, comorbidities and occurrence of bone union or complications were also recorded.

### Definitions

*Bone union* was defined as obliteration of the fracture line with presence of radiologically visible trabeculations across the fracture after successful healing [17].

*Pseudoarthrosis* was defined as absence of trabeculations across the fracture line later than 6 months post operation with/without redislocation of the fragments and/or painful weight-bearing [18–20].

*Fixation failure* was defined as loss of fixation resulting in displacement of the fracture within the first 2 months post operation [19].

*Nonunion* was defined collectively as pseudoarthrosis or fixation failure; as in the literature, the former often describes these two conditions constituting the main causes for re-operations [19, 21, 22].

### Parameters of interest

The following radiographical outcomes were investigated: bone union, nonunion and AVN. In addition to evaluation of the clinical results by Harris HS, three important indicators for independent living were also evaluated: relief of pain (good, poor), mobility (good, poor) and putting on socks and shoes skills (easy, difficult) [5].

The number of existing comorbid diseases was also considered for evaluation.

### Patient groups

Aiming to achieve comparability, patient grouping was performed according to age, gender, fracture displacement (stage Garden III or IV), and patient status prior to fracture in terms of comorbid diseases (less than one, two or more) [5]. Applying the above-mentioned criteria for gender and number of comorbidities, the patients were divided into four main groups as given in Table 1. Groups based on patients’ age are presented in Table 2. Patients aged 66 years or more were divided into age groups of 5 years each. In addition, patients older than 66 years with two or more comorbidities were evaluated separately.

**Table 1:** Patient groups according to gender and number of comorbidities

Group	Description	Patients
1	Males with two or more comorbidities	42
2	Males with up to one comorbidity	0
3	Females with two or more comorbidities	161
4	Females with up to one comorbidity	4

**Table 2:** Age groups of patients

Age groups (207 patients)								
Age, years	≤65	66–70	71–75	76–80	81–85	86–90	91–95	96–100
Patients	29	20	32	48	44	29	4	1
Percentage	14.00%	9.66%	15.45%	23.18%	21.25%	14.00%	1.93%	0.48%

### Statistical analysis

Statistical analysis was performed with the use of SPSS software package (Version 21, IBM, Armonk, NY, USA). After screening the normal distribution with Shapiro–Wilk test, Mann–Whitney test with Bonferroni correction for multiple comparisons was applied to detect significant differences in age and Harris HS distributions. In addition, Fischer’s exact test was used to screen significances in frequency distributions between gender, Garden type fracture, bone union, AVN, relief of pain, mobility, putting on socks and shoes skills, and comorbidities. The level of significance was set to *P* = 0.05 for all statistical tests.

## Results

The subjected patients comprised 42 males (20.28%) and 165 females (79.71%), aged 75.7 ± 10.3 (range 49–99) and 76.4 ± 9.8 (range 38–93) years, respectively, with no significant age difference between the genders (*P* = 0.56).

The average follow-up period was 29.6 months (range 12–78), including all complications. Five complications were developed within less than 12 months. Moreover, 100, 79.7, 53.1 and 22.9% of the patients were followed-up postoperatively after 12, 18, 24 and 36 months, respectively.

### Radiographical results

**Fig. 2:**
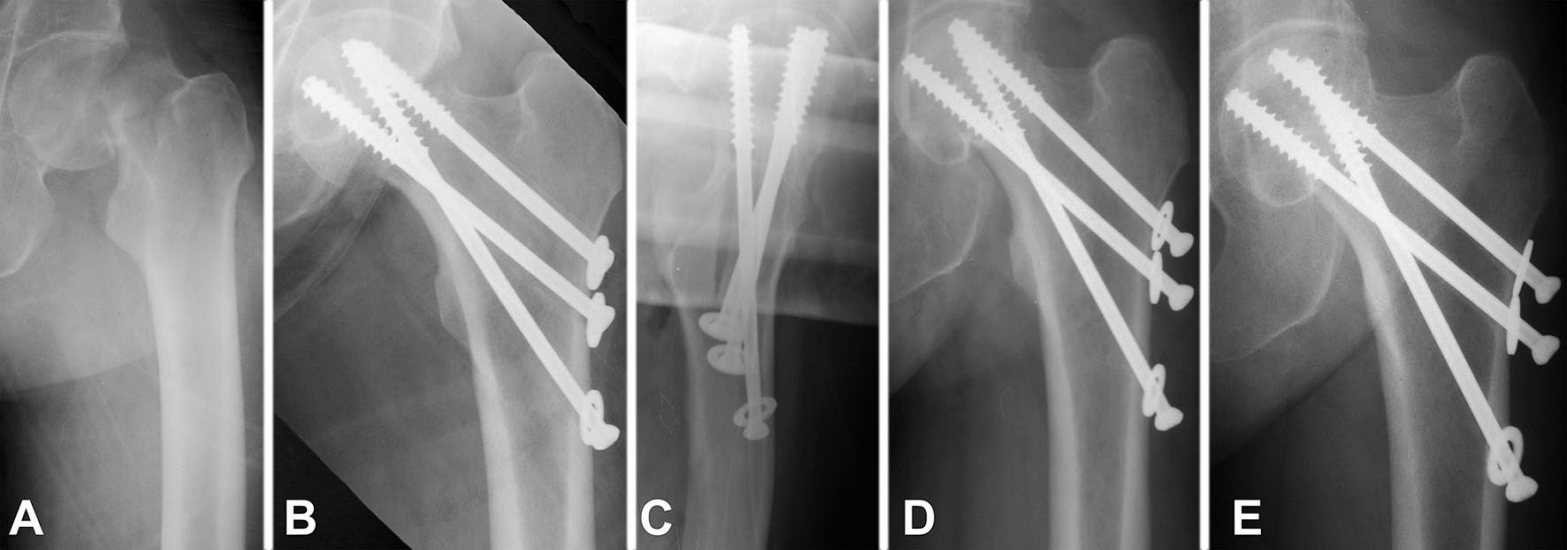
Exemplified X-rays of a 51 yo female patient with bone union (successful healing with fracture consolidation) after BDSF treatment: AP view diagnostics (**a**), postoperative AP view (**b**), postoperative lateral view (**c**), AP view 6-month follow-up (**d**) and AP view 55-month follow-up (**e**)

**Fig. 3:**
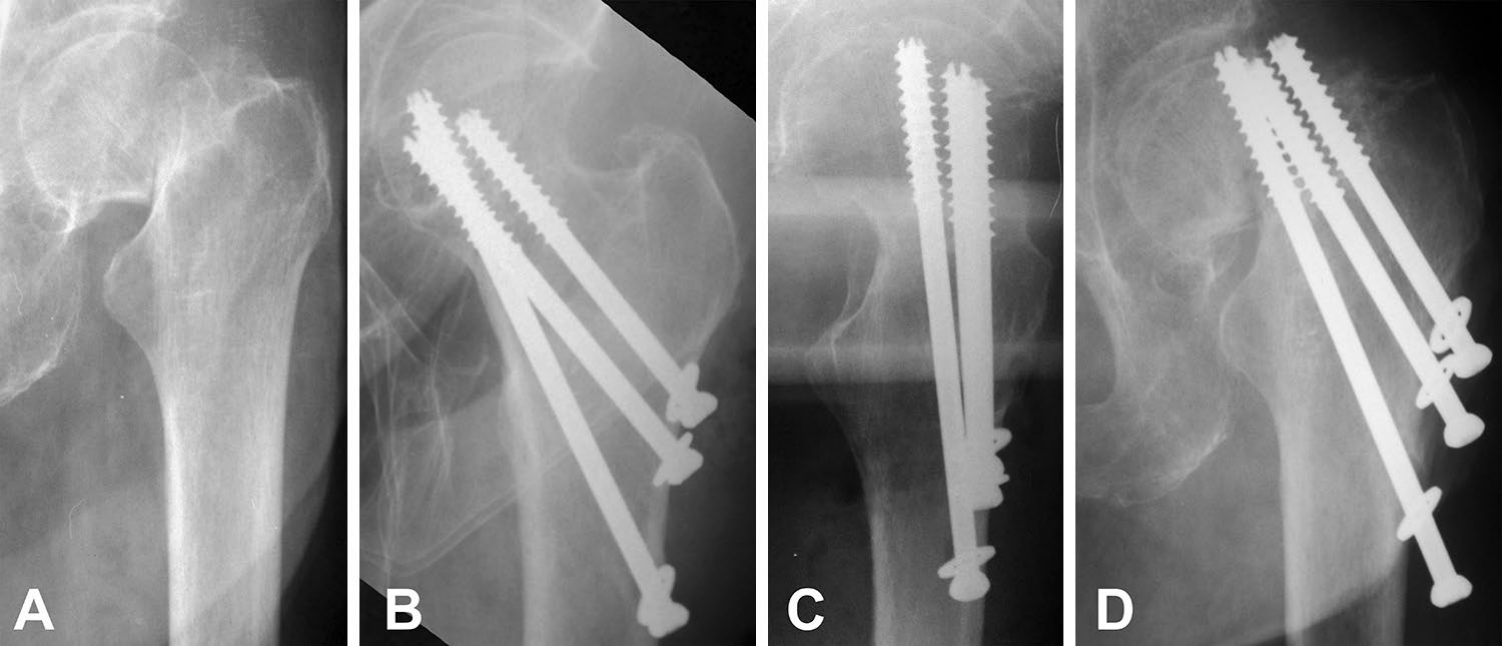
Exemplified X-rays of a 74 yo female patient with complete fixation failure (fragment redislocation) after BDSF treatment: AP view diagnostics (**a**), postoperative AP view (**b**), postoperative lateral view (**c**) and AP view 2-month follow-up (**d**). Lack of posterior cortical support and malreduction are seen on the postoperative X-rays (**b, c**)

**Fig. 4:**
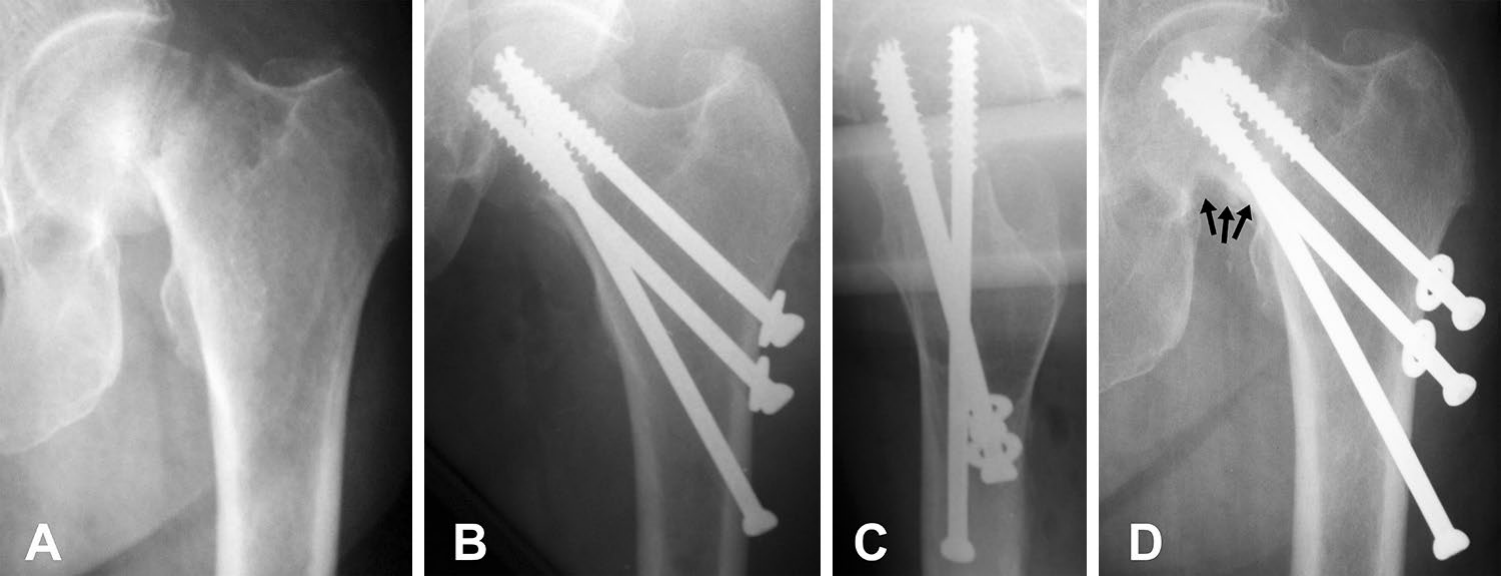
Exemplified X-rays of a 73 yo female patient with incomplete fixation failure and fragment impaction as a result of extreme loading after BDSF treatment: AP view diagnostics (**a**), postoperative AP view (**b**), postoperative lateral view (**c**), AP view 20-day follow-up with incomplete fixation failure and excessive fragment impaction, the latter denoted by *black arrows* (**d**). This patient died of acute renal insufficiency. Although severe traumatic agent caused partial displacement, the proper positioning of the screws prevents total displacement and transforms the shearing forces into compressive, causing further impaction

**Fig. 5:**
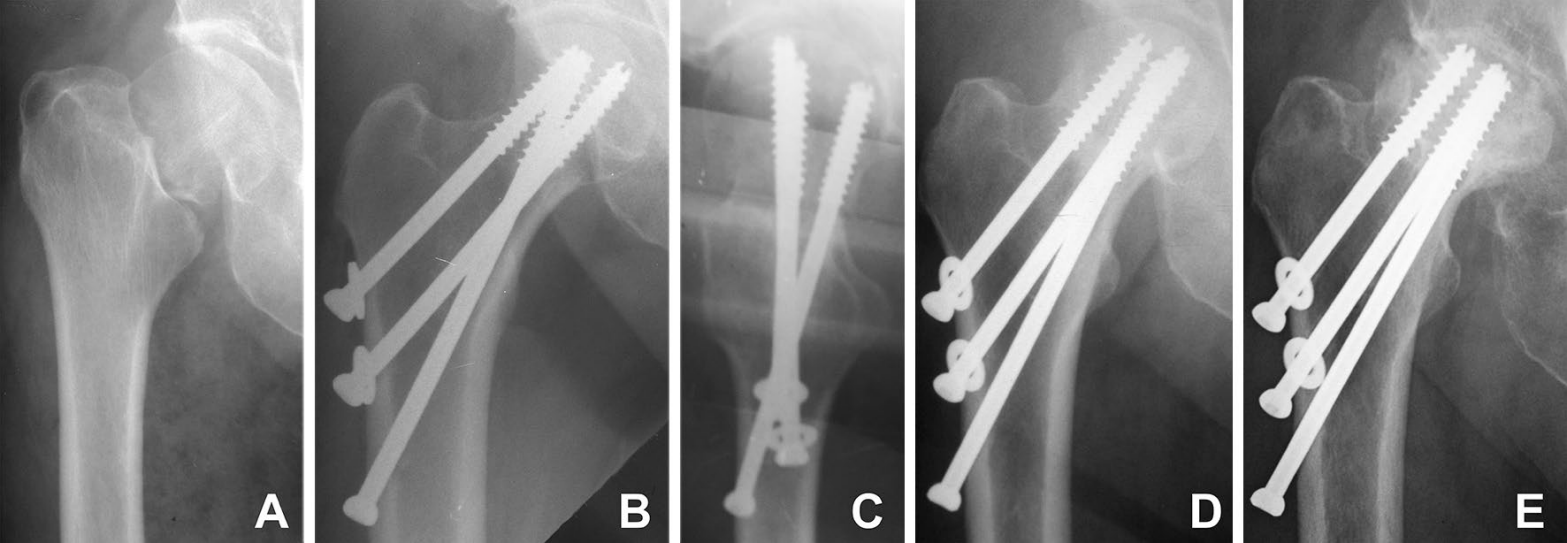
Exemplified X-rays of a 79 yo female patient with late AVN post bone union after BDSF treatment: AP view diagnostics (**a**), postoperative AP view (**b**), postoperative lateral view (**c**), AP view 10-month follow-up with bone union (**d**) and 24-month follow-up with late AVN (**e**)

Radiographical results of the current study are presented in Table 3. The registered rate of bone union (Fig. 2) was 97.6% in males (41 out of 42 cases) and 96.4% in females (159 out of 165 cases). One case with pseudoarthrosis was registered in a 68 years-old (yo) male after Garden IV fracture and 1 nonunion with AVN developed in an 80 yo female with full resorption of the femoral head after a Garden IV fracture. Fixation failure occurred in 5 females older than 65 years only: 2 of the cases were with complete fixation failure (fragment redislocation, Fig. 3) and the rest of 3 cases were with incomplete fixation failure as shown in Fig. 4, where excessive impaction of the fragments was observed as a result of extreme loading, however, with minimal fracture displacement of less than 3 mm. One case of non-iatrogenic subtrochanteric fracture occurred in an 84 yo male after successful healing two months post operation, despite properly positioned screws. AVN was developed in 12.1% of all patients (25 out of 207 cases). All cases with AVN except one case were developed post bone union and were classified both as AVN and as bone union (Fig. 5) [17, 21, 23].

**Table 3:** Bone union, nonunion and AVN distribution among all 207 patients, as well as among 178 patients older than 66 years with two or more comorbidities

Patients	Bone union	Nonunion				AVN post union	AVN total
		Fixation failure	Pseudoarthrosis	Nonunion with AVN	Total		
All (*n* = 207)	96.6% (200/207)	2.4% (5/207)	0.5% (1/207)	0.5% (1/207)	3.4% (7/207)	11.6% (24/207)	12.1% (25/207)
Older than 66 years, with two or more comorbidities (*n* = 178)	96.1% (171/178)	2.8% (5/178)	0.6% (1/178)	0.6% (1/178)	4.0% (7/178)	12.9% (23/178)	13.5% (24/178)

*AVN* avascular necrosis

### Clinical and functional results

Good functional outcomes were registered in majority of the cases, as presented in Table 4.

**Table 4:** Functional outcomes—distribution among all 207 patients

Pain relief		Mobility		Putting on socks and shoes		Harris HS			
Good	Poor	Good	Poor	Easy	Difficult	Excellent (90–100)	Good (80–89)	Fair (70–79)	Poor (10–69)
88.4%	11.6%	83.6%	16.4%	80.7%	19.3%	60.4%	17.9%	10.1%	11,6%

*Harris HS* modified Harris hip score

### Relation between age, gender, radiographical, clinical, and functional results

#### Patient age

Data for the relation between age and other radiographical, clinical, functional results and gender of the patients are given in Table 5.

**Table 5:** Patients’ age and Harris HS, given in terms of mean value ± standard deviation and range, in relation to gender, fracture type, radiographical, clinical and functional results, together with *P* values from comparisons within each category

	Gender		Garden Type		Bone union		AVN		Pain relief		Mobility		Putting on socks and shoes		Comorbidities	
	Female	Male	III	IV	Yes	No	No	Yes	Poor	Good	Poor	Good	Difficult	Easy	≤1	≥2
Age	76.4 ± 9.8 (38, 93)	75.7 ± 10.3 (49, 99)	73.9 ± 9.5 (52, 84)	76.4 ± 9.9 (38, 99)	76.1 ± 9.9 (38, 99)	82.2 ± 7.3 (73, 93)	76.1 ± 10.3 (38, 99)	77.7 ± 5.6 (62, 88)	78.1 ± 6.5 (62, 93)	76.0 ± 10.2 (38, 99)	80.8 ± 5.9 (68, 93)	75.4 ± 10.2 (38, 99)	81.3 ± 6.7 (63, 99)	75.1 ± 10.1 (38, 93)	55.8 ± 3.4 (51, 59)	76.7 ± 9.5 (38, 99)
*P* value	0.56		0.30		0.21		0.86		0.71		0.005		<0.001		0.001	
Harris HS	85.4 ± 19.1 (10, 100)	89.3 ± 18.1 (18, 100)	94.0 ± 9.1 (71, 100)	85.6 ± 19.4 (10, 100)	88.2 ± 15.6 (18, 100)	27.1 ± 19.3 (10, 57)	90.2 ± 14.5 (10, 100)	56.8 ± 21.5 (18, 88)	42.8 ± 20.5 (10, 85)	91.9 ± 8.5 (65, 100)	52.8 ± 23.4 (10, 82)	92.7 ± 7.9 (61, 100)	58.9 ± 24.9 (10, 91)	92.7 ± 8.7 (44, 100)	100	85.9 ± 18.9 (10, 100)
*P* value	0.07		0.04		<0.001		<0.001		<0.001		<0.001		<0.001			

*AVN* avascular necrosis, * Harris HS* modified Harris hip score

No statistical significance was found when relating patients’ age to gender, degree of fracture displacement, bone union and relief of pain (*P* ≥ 0.21). Older patients were admitted with significantly more comorbidities (*P* = 0.001), and on follow-up, they were significantly less mobile (*P* = 0.005) and had significantly more difficulties to put socks and shoes on (*P* < 0.001).

In addition, age of the patients with excellent Harris HS was significantly lower than those with either poor, fair or good Harris HS (*P* ≤ 0.04). Moreover, bone union and pain perception rates were similar among the 7 age groups of patients (*P* = 0.33 and *P* = 0.36, respectively). In contrast, although AVN incidence (yes/no) was not significantly related to patients’ age (*P* = 0.86), the AVN rate was significantly different among the 7 age groups (*P* = 0.02) with the highest incidence in the age group 76–80 years—27.1% of all cases and 30% among females. Similarly, the rate of mobility and putting on socks and shoes skills was significantly different among the 7 age groups (*P* = 0.02 and *P* = 0.04, respectively).

#### Modified Harris hip score

Among all 207 patients, the Harris HS was 86.2 ± 18.9 (range 10–100), with no significant difference between genders, *P* = 0.07 (Table 5). This score was significantly higher for patients with Garden III versus Garden IV fractures (*P* = 0.04), and after bone union in comparison to cases with nonunion (*P* < 0.001). On the other hand, Harris HS was significantly lower for patients with AVN compared to those without AVN (*P* < 0.001), for patients with poor versus good relief of pain (*P* < 0.001), as well as in cases with poor versus good mobility (*P* < 0.001) and for patients declaring difficult versus easy putting on socks and shoes skills (*P* < 0.001).

Harris HS for patients aged below 65 years was similar to the age group 66–70 years, but significantly higher than in all other age groups (*P* ≤ 0.04).

#### Gender

Fracture type (Garden III or IV), bone union (yes or no), as well as number of comorbidities were with no significantly different incidence between the genders (*P* ≥ 0.52). However, following each of the 7 age groups separately by genders, the incidence of nonunion was much higher in the female group 91–95 yo (33%) versus all other age groups (0–6%). Female and male patients declared similar perception with regard to their pain, mobility and putting on socks and shoes skills (*P* ≥ 0.24).

#### Fracture displacement stage

The number of comorbidities was similar with the incidence between patients with either Garden III or IV fracture (*P* = 0.98). All cases with complications (nonunion, AVN) occurred after Garden IV fractures.

#### Avascular necrosis

Patients with AVN declared significantly more occasional pain, poor mobility and difficulties with putting on socks and shoes compared to patients without AVN (*P* = 0.001). The number of comorbidities was with similar incidence between patients with or without AVN (*P* = 0.99).

#### Functional results and comorbidities

The number of comorbidities was not related to patient perception with regard to pain, mobility and putting shoes skills (*P* = 0.98).

## Discussion

The current study is focused on the clinical and radiological outcomes in neck of femur fracture fixation using the biplane double-supported screw fixation method described by Filipov.

In respect of the fixation strength, the most original and effective in this method is the distal screw—placed at obtuse angle and supported on a large area along the distal and posterior cortex of the femoral neck following its spiral anterior curve. Thereby, BDSF realizes the strongest possible distal and posterior cortical support for the fixation construct. Furthermore, the two calcar-buttressed screws have their medial cortical supporting points located apart from each other, spreading the weight-bearing load over approximately 50% of the femoral neck cortex length without concentrating stress in a single spot. The steeper screw orientation contributes to increased varus resistance and allows for easier screw sliding, thus avoiding cut-out and maintaining stronger fixation strength. Moreover, the nonparallel orientation of the screws does not prevent their sliding in the femoral neck, which biomechanically represents a hollow cylinder.

We did not observe any iatrogenic subtrochanteric fracture after surgery, although the placement of the most distal screw is below the distal end of the lesser trochanter. Some previous studies conclude that the screws should not enter the lateral femur below the lesser trochanter to prevent this complication [13, 24]. Admittedly, the small distance of less than 7 mm between the three parallel cannulated screws, used in those studies, may be a significant stress-riser in this area. However, the rather wide distance between screws in BDSF (20–40 mm) might not weaken the subtrochanteric femur bone, because the tensile forces acting on the lateral cortex are spread over a larger area.

Entering the cortex in such an oblique angle is best performed using new, sharp 2.8-mm guide wires. Any thermal necrosis caused during driving the guide wire in the cortex is later eliminated with the subsequent reaming.

The period defined in the literature for occurrence of bone union after osteosynthesis of femoral neck fractures is usually within 3 months post operation, and all complications related to mechanical and/or biological deficiencies, called with the collective term nonunion, occur within 6 months, including failure of fixation and pseudoarthrosis [15, 18, 19, 21, 22]. Therefore, we assumed a minimal follow-up period of 12 months as sufficient to demonstrate occurrence of bone union or a complication of mechanical type.

It is reported that the quality of reduction is the single most important factor within the surgeon’s control influencing the rate of healing complications [25, 26]. In our study, beside the quality of reduction, it was found that the positions of the screws are also very important factors for fracture healing. Two out of the five cases of fixation failure were cases with complete fixation failure, where we found malposition of the screws in one of them and a significant lack of posterior cortical support and fracture malreduction in the second one (Fig. 3). And vice versa, only the proper screw position with cortical support was the factor that saved one of the incomplete fixation failure cases from its complete displacement (Fig. 4). Furthermore, all cases with complications (7 nonunions and 25 AVNs) were registered in patients with Garden stage IV fractures, older than 68 years, and all of them were females (except 3 complications occurred in males). This confirms the opinion that besides fracture reduction and screw position, there are three additional factors related to complication rate: degree of displacement, age and gender.

The literature data for conventional fixation methods report bone union rate of about 84% (range 54–82), with nonunion rate ranging from 18 to 46%, and particularly a rate of fixation failure ranging from 9 to 30% [1, 2, 5, 7, 24, 27, 28].

The rate of AVN seems to be similar worldwide and is slightly influenced by the applied fixation method, rating about 9% (range 6–19%) for undisplaced and about 16% (range 9–32%) for displaced fractures [3–6]. Conventional methods are related to moderate or severe pain in 30 to 43% of the patients, good relief of pain in about 70%, good mobility in 63% and poor mobility rate in 37% [2, 5].

With the higher rate of bone union (96.6%), lower incidence of nonunion (3.4%) and particularly fixation failure in only 2.4%, our data show that the method of BDSF demonstrates significantly better results compared to the literature data for conventional fixation. The registered in our study rates of AVN, pain, and mobility are comparable to that reported in the literature.

A limitation of the current study is that no computed tomography (CT) or magnetic resonance imaging (MRI) was applied for diagnosis of AVN. Also, despite the follow-up period of 29.6 months on average, the minimal follow-up period of 12 months may have been insufficient to register all cases of AVN. Further, although patient outcomes after fixation with the novel BDSF method were compared to literature data, there was no control group with application of three parallel cannulated screws in the current study. The reason for that was the insufficient number of patients operated with this conventional fixation technique during the retrospective period, due to its exclusion to use because of high failure risk.

Further to the results reported in our recently published biomechanical comparative study, the current clinical study reconfirms that the better outcomes following BDSF treatment are due to its high fixation strength [10].

With its very strong cortical support and increased screw angle, BDSF allows for immediate full weight-bearing, as it is reflected in the high Harris HS functional results and good independent daily living abilities of the patients.

In the recent years, there is observed a trend using more and more hemiarthroplasties in displaced femoral neck fractures, but we should not forget that more than 90% of these fractures may heal and in 85% the healing will be uneventful.

The BDSF method can be easy learned within a few applications under guidance; however, for rather unexperienced surgeons, it could be more difficult to achieve anatomical fracture reduction and proper C-arm image evaluation.

## Conclusion

By providing additional cortical support, the novel BDSF method enhances femoral neck fracture fixation strength, reveals excellent clinical outcomes and is a valid alternative to other treatment methods.
